# Impact of Parents’ Attitudes on Learning Ineffectiveness: The Mediating Role of Parental Self-Efficacy

**DOI:** 10.3390/ijerph19010615

**Published:** 2022-01-05

**Authors:** Xiaohong Liu, Li Zhao, Yu-Sheng Su

**Affiliations:** 1 School of Education Science, Nanjing Normal University, Nanjing 210097, China; 210601019@njnu.edu.cn (X.L.); li.zhao@njnu.edu.cn (L.Z.); 2 Department of Computer Science and Engineering, National Taiwan Ocean University, Keelung 202301, Taiwan

**Keywords:** online learning, learning ineffectiveness, parental self-efficacy, mediator analysis, school-family cooperation

## Abstract

Parents play a key role in children’s home-based online learning. This study constructed a mediating model to explore the mechanism of parents’ attitudes toward online learning (PATOL) and the perceived online learning ineffectiveness (POLI) of their children and to investigate the mediating effect of parents’ self-efficacy (PSE) on PATOL and POLI. Valid questionnaire data from 18,170 middle school parents were collected by snowball sampling. The hypotheses proposed in this study were verified by using Model 4 of PROCESS. The results showed that: when controlling parents’ gender, age, and children’s length of online learning in regression equations, (1) both PATOL and PSE were negatively related to POLI, while PATOL was positively related to PSE; (2) PSE played a mediating role in the relationship between PATOL and POLI. This study also discusses how to support parents to assist children’s home-based online learning. Schools should carry out some necessary training for parents. Parents can get guidance and advice on how to create an environment conducive to children’s online learning.

## 1. Introduction

Many countries have adopted home-based online learning instead of face-to-face learning to reduce the spread of COVID-19 [1]. The shift from offline school learning to home-based online learning resulted in changes in the learning environment and amplified the parents’ role in children’s schoolwork [2]. During the epidemic, home-based online learning can prevent the spread of the virus in physical spaces [3], and it makes parents feel at ease because their children are in a safe environment at home. However, low student engagement, participation, and motivation during online learning have been found among students [4]. In particular, students’ mental disorders and psychological problems are also aroused in home-based online learning [5,6]. Thus, without the teacher’s face-to-face instruction, parents need to take on greater responsibility for academic, emotional, and technical support of their children’s home-based online learning [7]. For example, parents need to provide learning guidance, motivate and monitor their children’s progress, and help to develop the skills to manage learning time and learning persistence [8,9]. In addition, the mental health of students during an epidemic lockdown is a topic of concern; however, mental health issues are not yet at the forefront of existing policies [10,11,12]. During the lockdown, most students could only receive online learning at home, and parents, as guardians, provided great help for the implementation of their online learning. Therefore, many studies also called for the support and help of parents for the mental health problems of children learning online at home [12]. Saha and Sifat [10] suggested that paying attention to the effectiveness of students’ online learning could prevent students from developing serious mental health problems. In total, successful distance online learning requires parental support [13]. Thus, it is very important to know parents’ attitudes and evaluation of online learning for school-family cooperation in online learning. Given the development of the global pandemic, Kissler et al. [14] stated that schools should still prepare for repeated school suspensions in the next 2 years. However, few studies have focused on parents’ views regarding online learning at home during COVID-19 [15,16], especially, the evaluation of the implementation process of online learning. Thus, this study aims to explore online learning from the perspective of parents to fill this gap. It can provide suggestions for the formation of a good school-family cooperation model.

Compared with face-to-face learning, parents encounter more difficulties or problems supporting their children’s online learning and feel greater parenting pressure [16,17,18]. Parental stress was found to be negatively related to their provision of home online learning activities [19]. Parents’ attitudes toward online learning and their perceived online learning effectiveness during children’s home-based online learning may influence their engagement in their children’s education, and thus have negative effects on their children’s online learning quality [20]. Thus, parents’ attitudes toward online learning may be related to their children’s online learning effectiveness. Previous studies have explored parents’ satisfaction with online learning [21], parents’ attitudes and beliefs about online learning [18] and parents’ involvement in their children’s online learning [9]. However, the relationship between parents’ attitudes toward online learning (PATOL) and perceived online learning effectiveness has not yet been discussed, although a definition of perceived online learning ineffectiveness (POLI) was proposed by Hong et al. [22], and was used to evaluate the negative online learning effectiveness during the lockdown. The study hypothesized that PATOL was negatively related to POLI.

During the pandemic, health concerns were not a significant predictor of perceived stress among parents, but rather the consequence of lockdowns [16,23]. Lockdowns have exacerbated existing pressures on parents, such as housing pressures and family conflicts. In addition, they have created additional pressures, such as unemployment and taking care of children’s studies while working at home [23]. According to the family stress model, the increase in parents’ pressure has led to a decrease in their learning involvement behaviors, and less parental involvement in their children’s learning could mean lower levels of cognitive or emotional resources provided to the children [19]. Parents’ self-efficacy (PSE) is a protective factor that alleviates the negative impact of stress, so it may interfere to some extent with the negative impacts of various pressures on their family education activities. For example, some studies have shown that parents with high levels of PSE have more confidence in their ability to cope with difficult parenting situations and to promote children’s learning with lower levels of negative emotion [19,24]. During the pandemic, PSE may play a protective role in the relation between PATOL and POLI. Therefore, this study aimed to construct a mediation model to investigate the relationship between PATOL, PSE, and POLI. The mediation role of PSE was examined. The findings offer some ideas for schools to organize home-based online learning.

## 2. Theoretical Background and Hypotheses

### 2.1. Parents’ Attitudes toward Online Learning and Perceived Online Learning Ineffectiveness

Parents’ attitudes toward online learning (PATOL) refer to parents’ evaluation of online learning [18]. Generally, parents have both negative and positive attitudes toward online learning. Parents recognize that online learning has positive effects on children’s language skills, technical skills, and learning abilities [25]. However, some parents believe that online learning is time-consuming and burdensome, and may affect their children’s mental and physical health development (e.g., the deterioration of eyesight, Internet addiction) [18,26]. Although parents have mixed opinions on online learning, they still participate in their children’s online learning [27]. Dong et al. [18] developed a PATOL measurement to evaluate online learning in terms of its value, pros and cons, and its impact on family education. During home-based online learning, parents are also concerned about the efficiency of student–teacher interactions, the interest and learning state of their children during online learning, and whether their children can understand the learning content [28]. Therefore, in this study, PATOL refers to parents’ attitudes towards online learning compared with face-to-face learning and perceived evaluation of the online learning implementation process.

Perceived online learning ineffectiveness (POLI) refers to learners’ negative evaluation of learning efficiency, concentration, learning state, and other aspects after switching from face-to-face school learning to home-based online learning [22]. Previous studies related to the effectiveness of K-12 students’ online learning are limited, and the results are not consistent [29]. During COVID-19, the effectiveness of online learning also remains debatable compared to face-to-face learning [30]. Online learning has achieved positive results during the pandemic, with both students and teachers holding positive attitudes towards it [31]. Although most students are satisfied with online learning, students prefer face-to-face learning, due to the influence of network availability, network connectivity, student–teacher online interaction, and other factors [32]. At present, research on the effectiveness of online learning has mostly been conducted from the perspectives of students and teachers, while parents’ perspectives have not been sufficiently discussed. Parents’ attitudes toward online learning have an influence on their involvement behavior in their children’s learning activities and online learning quality [19,20]. Moreover, parental involvement has a positive impact on student outcomes [33]. Thus, this study proposed the hypothesis (H1): PATOL is negatively related to POLI.

### 2.2. The Mediating Effects of Parental Self-Efficacy

Self-efficacy is conceptualized as a belief in one’s ability or skill of accomplishing something [34]. According to the competence of self-efficacy and social cognitive theory proposed by Bandura [35], parental self-efficacy (PSE) refers to the individual’s belief in promoting the development of their children and their positive outcomes [34]. Thus, PSE is always related with parental educational practices. For example, parents with high PSE levels are more likely to adopt educational strategies and behaviors that promote their children’s social and academic learning [36].

In the online learning environment, it is more necessary for parents to assist their children in dealing with the problems encountered in online learning, and even to shoulder part of the instructional role [8,37]. Parents with higher PSE levels tend to be more engaged in their children’s learning and to provide a better quality of family learning environment for their children, such as creating learning opportunities every day for children, increasing parent-child interaction, and making learning resources more accessible [19,38]. Negative emotions (e.g., parents’ pressure) lead to a decrease in parents’ home-based online learning behavior, and PSE, as a protective factor, can alleviate parents’ pressure to participate in their children’s home-based online learning [19]. Moreover, parents with higher PSE levels are more confident in their ability to support children’s development and learning and are more likely to cope with difficult parenting challenges with lower negative emotions [24]. Liu and Leighton [39] also found that positive perceptions (e.g., specific teacher invitations and general school invitations) may promote PSE. Thus, this study proposed hypothesis (H2a): PATOL is positively related to PSE. Moreover, adolescents’ perceived parental support was positively correlated with their academic value and learning motivation [40]. When children perceive higher parental support, their learning motivation is correspondingly encouraged, leading to better learning performance. Thus, higher-level PSE can predict the success of children’s online learning performance. For example, Liu and Leighton [39] found that PSE was positively related to children’s mathematics achievement. Therefore, this study proposed the hypothesis (H2b): PSE is negatively related to POLI.

PSE can directly affect children’s adaptive ability, but also indirectly affect their adaptive ability through their parents’ engagement behavior [41]. In the transition from offline to online learning, parents’ PSE is helpful for children to adapt to the change in learning environment. As discussed above, parents with high PSE enhance their children’s learning with lower negative emotions and have confidence in coping with difficult parenting situations [19,24]. It is an important factor for children’s development that parents provide a family learning environment with cognitive stimulation. Liu and Leighton [39] showed that PSE mediated the relationship between parents’ positive perceptions (e.g., specific teacher invitations and general school invitations) and children’s achievement. Oppermann et al. [19] also found that parents’ self-efficacy plays a mediating role in parents’ negative emotions (e.g., parental stress) and parenting practice behaviors, which can alleviate the negative impact of parents’ emotions on parenting practice behaviors. However, the mediating role of PSE between PATOL and online learning ineffectiveness has not been discussed. Thus, this study proposed hypothesis (H2): PSE mediates the relationship between PATOL and POLI.

### 2.3. Hypothesized Conceptual Model

**Figure 1:**
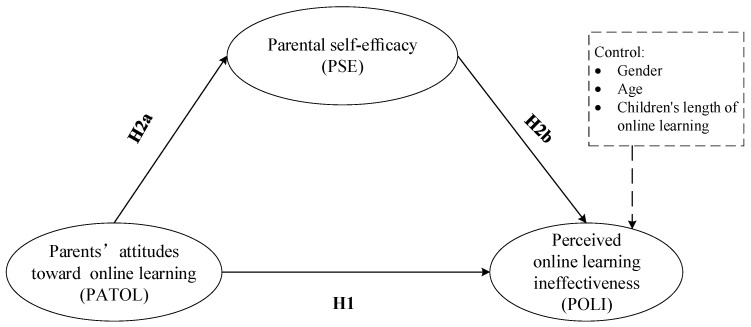
Hypothesized conceptual model.

Based on the above discussion, during the pandemic lockdown, many students have had to study online at home. It is difficult for students to adapt quickly to the change of learning environment. Moreover, confinement to home while studying may cause children’s mental health problems [5,6]. Thus, parents engage in their children’s home-based online learning, and also take on more supervision tasks than in offline learning [2,7]. Existing studies related to home-based online learning during the pandemic also call for parents to participate in their children’s learning and create a good learning environment for their children (e.g., [42]). Parents’ attitude towards the implementation process of online learning and their evaluation of their children’s online learning effectiveness also affect their participation in children’s online learning behavior [20]. However, few studies have explored parents’ perceptions of the online learning implementation process during the pandemic. As a protective factor of negative effects, parental self-efficacy may alleviate parents’ negative evaluation of online learning [24]. Thus, we constructed a mediation model to examine parental self-efficacy as a mediator in the relationship between PATOL and POLI. Parents’ gender, age, and children’s length of online learning were used as control variables when conducting the mediating effect test based on previous research [18,43]. It was shown that the hypotheses proposed in this study related to parental perceived online learning ineffectiveness had explanatory power beyond these three control variables. The hypothesized conceptual model proposed in this study is shown in Figure 1. The hypotheses were proposed as follows:

**Hypothesis 1** **(H1).** *PATOL is negatively related to POLI.*

**Hypothesis 2** **(H2).** *PSE mediates the relationship between PATOL and POLI.*

**Hypothesis 2a** **(H2a).** *PATOL is positively related to PSE.*

**Hypothesis 2b** **(H2b).** *PSE is negatively related to POLI.*

## 3. Methodology

### 3.1. Participants

Participants were the parents of middle school students from Jiangsu province, China. During the lockdown of COVID-19, most of the children in this province used the same official platform provided by the Provincial Department of Education to learn online. They watched the video, carried out online live instruction, and downloaded learning materials from the platform. Other tools, such as QQ and WeChat, were also used as supplementary tools.

### 3.2. Online Platform for the Questionnaire 

An online questionnaire was distributed on the online platform named *Questionnaire Star* (https://www.wjx.cn/ (accessed on 1 September 2021) that can restrict IP access to avoid respondents repeating questionnaires. Participants can open the online questionnaire by clicking a link via mobile devices, and terminate or quit at any time when filling in the questionnaire. Snowball sampling was adopted to collect data. The link of the online questionnaire was sent to 20 teachers from different middle schools who were contacted to help distribute the questionnaire to parents via instant chat platforms (e.g., WeChat or QQ). At the same time, we also asked these teachers to help send the link to other middle school teachers in Jiangsu province they knew to distribute the questionnaire.

### 3.3. Instrument

The online questionnaire consisted of three parts. In the first part, we stated that this survey was being conducted voluntarily and anonymously. The answers to the questionnaire were only available for the researchers and not for commercial or any other use. The second part was to collect the participants’ basic information. The third part was a scale designed for the variables in the study. The scale of the questionnaire used in this study was adapted from previous studies. The original scale was developed in English; thus, all items were translated into Chinese following the translation-back-translation procedure [44]. To ensure semantic equivalence, two Chinese bilingual academics translated all items into Chinese separately and individually translated them back into English. Two experts reviewed all items to ensure face validity. In addition, to ensure the readability of all items, three middle school students’ parents were invited to read all items and give feedback. All items were rated based on a 5-point Likert scale from “strongly disagree” to “strongly agree”. The questionnaire included 30 items. According to Zhan et al. [45], some volunteers were asked to answer the questionnaire. It was found that the questionnaire should be finished in at least 3 min. Therefore, if the response time of a questionnaire was less than 3 min, it was considered as a short response and was excluded from the analysis.

#### 3.3.1. Parents’ Attitudes toward Online Learning

This scale was adapted from Dong et al. [18] to assess parents’ perspectives on online learning from aspects such as the content, efficiency, and learning atmosphere of online learning. Nine items were designed to evaluate participants’ PATOL. Sample items include: “I think that online learning allows my child to study and review instructional videos over and over again” and “During online learning, although the instructor and the student are not in the same physical space, children’s questions can still be solved in time”.

#### 3.3.2. Parental Self-Efficacy

Seven items were used to measure PSE to understand parents’ confidence in supporting their children’s development in online learning. This study adopted the PSE scale developed by Purssell and While [46] to reflect parents’ feelings of problem-solving ability and competence. Sample items are: “Before class, I will pay attention to whether my child’s learning environment is quiet, to avoid irrelevant factors affecting their online learning” and “I will urge or help my child to check the stability of the computer, iPad and other related devices before class”.

#### 3.3.3. Perceived Online Learning Ineffectiveness

This scale has nine items to access participants’ POLI compared with face-to-face learning based on their children’s learning performance. The original scale developed by Hong et al. [22] was used to gauge students’ perceived performance during online learning. Sample items are: “Since online learning, my child’s learning efficiency has decreased significantly” and “My child’s academic performance has dropped significantly since online learning”.

### 3.4. Reliability and Validity Test of Scales

The reliability and validity of the measurement scale were subsequently evaluated. Confirmatory factor analysis (CFA) was used to establish the internal validity of each construct. Items with high residual value (>0.5) and low standardized factor loadings (<0.5) needed to be deleted [47]. Thus, three items were deleted from the PATOL scale, three from the PSE scale, and four from the POLI scale. CFA showed that the modified model fit the data well: RMSEA = 0.054 < 0.08, GFI = 0.964 > 0.9, AGFI = 0.951 > 0.9, and NFI = 0.978 > 0.9 [47,48]. Then, internal consistency reliability and composite reliability (CR) were evaluated (shown in Table 1). The Cronbach’s α values of each construct were all above 0.7, indicating acceptable reliability [48]. Moreover, the CR value of each construct surpassed 0.7, showing good composite reliability [48]. Regarding convergent validity, it can be evaluated by average variance-extracted (AVE) and factor loadings (FL). The values of AVE and FL of each construct were higher than 0.6, indicating an acceptable level of convergent validity (see Table 1) [48]. In addition, the skew and kurtosis of all items ranged from −1.23 to 0.932 and −0.748 to 1.751, respectively. Thus, the collected data had good distribution due to skew (<1.0) and kurtosis (between −2.00 and 2.00), all meeting their thresholds [49].

### 3.5. Common Method Bias

The data for all constructs were collected simultaneously through a self-reporting questionnaire; thus, common method bias (CMB) was a potential problem. CMB can be tested by Harman’s one-factor test and the CFA marker variable approach [50,51]. The result of Harman’s one-factor test indicated that the single factor accounted for 29.291% of the covariance amongst the model indicators, which is less than the recommended threshold (50%) [51]. Then, CMB in the model estimates was tested by the CFA marker variable approach via AMOS [50]. The CFA Model is a fully correlated model containing a Marker Variable. If there is no serious CMV, the correlation with potential variables is not too great [52]. The result showed that the correlation coefficient between PATOL and the Marker Variable, PSE and the Marker Variable, and POLI and the Marker Variable was 0.01, 0.00, and −0.01, respectively. PATOL, PSE, and POLI were not significantly correlated with the Marker Variable. Thus, CMV was not a serious problem in our data and was unlikely to contaminate the results.

### 3.6. Data Analysis

SPSS (IBM, Armonk, NY, USA) and AMOS (IBM, Chicago, IL, USA) were used to analyze the data. First, CFA was tested to ensure construct reliability and validity among variables. Second, the descriptive statistics were calculated. Third, to explore the potential relationships of the three variables, Pearson’s correlation analyses were conducted among PATOL, PSE, and POLI. Fourth, PROCESS as a macro of SPSS developed by Hayes [53] has been widely used to test mediation models in many studies (e.g., [54,55]). This study used PROCESS template model 4 to test the hypothesized conceptual models. It provides confidence intervals of the bootstrap test of the conditional direct and indirect effects. If zero is not included in the confidence interval values, the direct and indirect effects can be considered significant.

## 4. Results

### 4.1. Descriptive Analysis

A total of 23,096 parents responded to the questionnaire in one month from 1 July to 1 August 2020. After deleting questionnaires with an overly short response time (less than 3 min), those with answers that tended to be consistent and those of participants whose children had not received online learning, 18,170 valid questionnaires remained, giving an effective recovery rate of 78.67%. Participants were 12,500 males (68.8%) and 5670 females (31.2%). Most participants were aged between 36 and 40 years (M = 7913, 43.5%). In terms of educational level, 6220 (34.2%) and 6059 (33.3%) participants had high school certificates and Bachelor’s degrees or College degrees, respectively. Most participants’ children spent more than 4 h per day on online learning (M = 9408, 51.8%).

### 4.2. Correlational Analysis

ANOVA was used to examine the different groups of gender (male and female) and age (31–35 years, 36–40 years, 41–45 years, ≥46 years) differences in the three variables (PATOL, PSE, and POLI). The results showed that age had an effect on PATOL [*F* (1, 18,168) = 3.97, *p* < 0.05], PSE [*F* (1, 18,168) = 23.58, *p* < 0.001], and POLI [*F* (1, 18,168) = 21.20, *p* < 0.001]. Female parents had higher levels of PATOL, PSE, and POLI. Participants’ educational level also had an effect on PATOL [*F* (3, 18,166) = 3.63, *p* < 0.05], PSE [*F* (3, 18,166) = 17.72, *p* < 0.001], and POLI [*F* (3, 18,166) = 13.353, *p* < 0.001]. The result of post hoc tests indicated that younger parents tended to report higher levels of PATOL, PSE, and POLI. The participant groups with different lengths of children’s online learning time (<1 h, 1–2 h, 2–3 h, 3–4 h, and >4 h) were also examined in the three variables via ANOVA. Participants with different lengths of children’ online learning time also had an effect on PATOL [*F* (4, 18,165) = 54.67, *p* < 0.001], PSE [*F* (4, 18,165) = 24.03, *p* < 0.001], and POLI [*F* (4, 18,165) = 5.85, *p* < 0.001]. The result of the post hoc tests indicated that parents tended to report higher levels of PATOL, PSE, and POLI if their children needed to spend long hours on online learning.

Table 2 illustrates the mean (*M*) and standard deviations (*SD*) of PATOL, PSE, and POLI, and the correlations among PATOL, PSE, and POLI. The results revealed that there was a significant correlation among PATOL, PSE, and POLI. PATOL was positively correlated with PSE (*r* = 0.525 **), and negatively related with POLI (*r* = −0.135 **). PSE was also negatively correlated with POLI (*r* = −0.132 **).

### 4.3. The Mediating Role of PSE in the Relationship between PATOL and POLI

**Figure 2:**
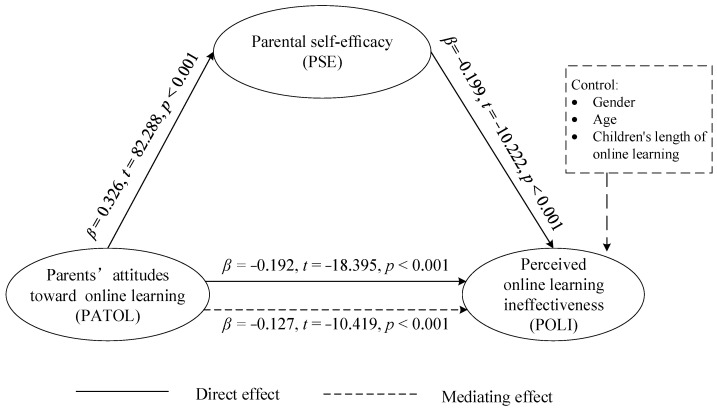
Mediating Role of Parental Self-Efficacy.

To test the hypothesis conceptual model, Model 4 of Hayes’s PROCESS macro was applied to conduct three regression models (see Table 3 and Table 4). Participants’ gender, age, and their children’s length of online learning were controlled in regression equations. The result also showed that PATOL was positively related to PSE (*β* = 0.326, *t* = 82.288, *p* < 0.001) and PSE was negatively related to POLI (*β* = −0.199, *t* = −10.222, *p* < 0.001). Thus, H2a and H2b were confirmed. The result showed that PATOL was negatively related to POLI (*β* = −0.192, *t* = −18.395, *p* < 0.001); thus, H1 was verified. The result of the mediating role of PSE is shown in Figure 2. Under the influence of PSE, PATOL was also negatively related to POLI (*β* = −0.127, *t* = −10.419, *p* < 0.001). Thus, PSE partially mediated the relation between PATOL and POLI, and H2 was also confirmed. The direct effect (−0.127) and indirect effect (−0.065) accounted for 66.15% and 33.85% of the total effect (−0.192) respectively.

## 5. Discussion

Online learning at home is a challenge for teachers, students, and parents due to the fact that some teaching strategies used in face-to-face learning environments cannot be directly applied to online learning environments [56,57]. Moreover, a quick network, technical support, and hardware facilities which are needed to support the online learning environment may not be available for every family. It is therefore difficult for instructors to solve the classroom organization problems and to evaluate students’ engagement state in time [3,58]. Thus, the children’s learning process is more dependent on parents than ever before [2]. Home-based online learning has changed teaching methods and has brought new challenges to school-family collaboration. This study investigated the relationship between PATOL, PSE, and POLI. This study also examined the mediating role of PSE between PATOL and POLI. The hypotheses proposed in this study were verified by 18,170 data from middle school students’ parents. Parents’ age, gender, and their child’s length of online learning were controlled in the model.

### 5.1. PATOL Is Negatively Related to POLI

The result of this study shows that PATOL was negatively related to POLI (*β* = −0.192, *t* = −18.395, *p* < 0.001, H1 supported), which is consistent with some existing studies (e.g., [19,20,33]). Those studies indicated that parents’ positive attitudes toward online learning can improve their engagement in child learning and child online learning quality, and thus have a positive impact on students’ outcomes. During home-based online learning, parents need to provide technical, emotional, and academic support for their children. One reason why parents held a negative attitude towards online learning is that they thought their children’s online learning, including the children’s health development, had not received enough attention from teachers [56,57]. For parents, perceived stress and negative parenting emotions were reduced when they perceived adequate support from school or teachers and perceived that their children’s learning was manageable [59]. Thus, schools can provide some suggestions to parents on how to manage time and mental health interventions for their children [29]. With regard to the online learning organizations, the number of unnecessary online learning platforms or software tools should also be reduced to alleviate parents’ anxiety [7,56]. To ease parents’ concern about their children’s eyesight declining, some alternative learning task activities could be provided to the children, such as some positive educational programming that is suitable for children to minimize excessive screen time [15].

### 5.2. The Mediating Role of PSE in the Relationship between PATOL and POLI

This study also confirms the mediation role of PSE between PATOL and POLI, and found that PATOL also related to POLI under the mediation of PSE (*β* = −0.127, *t* = −10.419, *p* < 0.001, H2 supported). Therefore, when parents had a positive evaluation of online learning activities provided by the school, their PSE was higher, which in turn enhanced their belief in assisting children’s home-based online learning. Parents are familiar with their children’s learning habits and can help to develop the concentration and resilience necessary for their children’s online learning [21]. They can support instructors and students in transitioning from offline face-to-face learning to home-based online learning. As a result, children can have better online learning performance.

#### 5.2.1. PATOL Is Positively Related to PSE

This study confirmed that PATOL was positively related to PSE (*β* = 0.326, *t* = 82.288, *p* < 0.001, H2a supported). This result is consistent with research conducted by Liu and Leighton [39] that positive cognition may promote PSE. If parents are satisfied with their children’s learning and engagement opportunities organized or provided by the school, they will have higher parental engagement and lower parenting stress [60]. PSE also reduces parenting pressure to participate in children’s home-based online learning, which in turn increases their PSE [19].

#### 5.2.2. PSE Is Negatively Related to POLI

Moreover, this study found that PSE was negatively related to POLI (*β* = −0.199, *t* = −10.222, *p* < 0.001, H2b supported). This result is similar to the findings recommended by previous studies (e.g., [19,38,39]) regarding parents’ high PSE having a positive effect on their provision of home learning activities for children and predicting their children’s better online learning performance.

Based on the above findings, it can be found that PATOL and PSE are very important in students’ online learning at home. When parents have a positive attitude toward online learning, they have higher levels of PSE and their children have better online learning performance. However, despite the increased need for parental involvement in K-12 students’ online learning, parents may not understand their important position in children’s online learning, and even when parents are aware of it, they also need to receive training and assistance continually [8]. For example, some parents were less confident about their ability to support children’s online learning. Moreover, they were even unable to create a structured online learning environment for their children [18,57]. Therefore, some appropriate support needs to be provided to parents by schools or teachers to promote parents’ PSE.

First and foremost, the government needs to provide some guidelines to guide schools to organize online learning and consider whether the learning content meets the educational requirements of students and parents without increasing their burden [15]. Plans for an emergency transition from face-to-face learning to distance home-based online learning also need to be prepared by the relative department [57], such as what techniques teachers use. Teachers should maintain long-term and high-quality communication with parents and convey those online learning plans to parents in a timely manner, to guide parents on how to create an environment conducive to online learning. Parents who are more fully informed about online learning also have reduced prejudice against online learning.

As for school, some training could be organized to help parents be made aware of how to strengthen their communication skills and daily activity planning skills, so as to enhance parents’ PSE. The content of such training can also cover information about children’s personality traits and self-regulated learning in the online learning environment, as well as differences between boys and girls [61,62]. Teachers also need to maintain timely communication with parents to know what aspects of support parents need most, so that parents can receive timely and effective help [15]. For example, the investigation of Ye et al. [29] found that both parents and students hoped that teachers could provide more feedback on homework and encourage students’ online learning to improve their motivation. In terms of homework assignments, parents also prefer their children to finish their homework in a group rather than individually, so as to strengthen the interaction between peers and to alleviate the loneliness. In an effective online environment, the teacher’s role is more to facilitate students’ learning than just to transfer knowledge [63]. In addition, the optimal level of social presence of teachers in online teaching is critical for students’ learning engagement [64]. Teachers strive to build such a supportive learning environment with a high level of social presence, strong interaction, collaboration, respect, and dependence, which can also provide learners with emotional support to help alleviate the loneliness of learners [12,65]. For example, the instructor can provide timely, specific, and constructive feedback in detail at different learning stages, or can allow students to take on more responsibility for their learning (e.g., discussion moderators) to enhance students’ motivation in the online learning process and to promote peer learning [65,66].

From the perspective of parents, they are concerned about the organization of online learning and the children’s online learning effectiveness. They also pay attention to children’s mental health problems caused during online learning [15,18,26,29]. In particular, school closures, strict lockdowns, daily indoor activities, participation in online courses, and reduced social relationships during COVID-19 lockdowns pose greater challenges for students’ mental disorders and psychological problems [5,6]. For example, Liu et al. [11] found that online learning inattention is positively correlated with anxiety and depression, and girls are more likely to show anxiety and depression than boys. Providing support for many aspects of children’s online learning can also solve students’ psychological distress [10]. Thus, other than parents, gatekeepers such as educators and even policymakers should also pay attention to modifiable factors of students’ mental health in home-based online learning, including psychological characteristics, family environment behaviors, and feelings.

## 6. Conclusions

The school-family collaboration has been influenced by the COVID-19 pandemic, and parents now play a more prominent role in tutoring and monitoring their children’s home-based online learning. The four hypotheses proposed in this study were supported to provide some suggestions for school-family cooperation during home-based online learning. PATOL was positively related to PSE (H2a) and negatively related to POLI (H1). PSE was negatively related to POLI (H2b). The mediator role of PSE between PATOL and POLI was confirmed (H2). Thus, it is beneficial to promote parents to establish their PSE. This study discusses how to provide the necessary support for parents to assist their children’s online learning. First, the government should formulate a plan in advance to deal with the emergency response to distance online learning in the future. Secondly, from the school’s perspectives, it is necessary to inform parents of online learning programs in time, so that parents are familiar with online learning programs and thus have a better understanding of online learning. Teachers also need to strengthen communication with parents, understand what kind of support parents need, and give parents some suggestions to assist their children’s learning (e.g., time management, mental health intervention, etc.).

### 6.1. Implications

Most of the published studies were conducted from the perspective of students or teachers to understand their perceived online learning effectiveness, but there is a lack of research from the perspective of parents. Additionally, there is no research discussing parents’ attitude toward the impact of the online learning implementation process on perceived online learning effectiveness. This study fills this gap. In the post-pandemic era, the investigation of online learning from the perspective of parents can help teachers and schools develop corresponding measures to provide some necessary support for parents, and then promote the cooperation between school and family.

This study has theoretical and practical contributions. First of all, this study confirmed the mediator role of PSE between PATOL and POLI. In this study, PATOL is more concerned with the evaluation of the implementation process of online learning, such as teacher-student interaction and the rationality of homework. When parents had positive attitudes toward online learning, their PSE was higher, and the self-efficacy negatively predicted POLI. In addition, parents with a positive attitude toward online learning also negatively predicted perceived online learning ineffectiveness. Thus, parents’ higher PSE and positive evaluation of the online learning activities help the school better carry out online learning and establish a positive school-family cooperation relationship. As for the practical value, the findings of this study have important guidance value for schools to implement online learning. Since the pandemic, parents have had to worry about the spread of the virus, and cope with the relationship between work and children’s studies. There is no doubt that taking on the responsibility of monitoring their children’s home-based online learning has increased parents’ anxiety levels. For schools, helping parents understand the advantages of online learning and providing some necessary support to improve their PSE in children’s online learning can facilitate the implementation of home-based online learning and reduce parents’ anxiety.

### 6.2. Limitations and Future Study

When interpreting the findings of this study, several limitations should be considered. First, the participants of this study were recruited from middle school students’ parents in Jiangsu, China, which may limit the generalizability of the research results. Future studies can recruit participants from central and western regions and even remote areas. In other words, the income of families may also influence the implementation of online learning to some extent [67]. This factor can be considered in future studies.

Second, a cross-sectional study was used to obtain data, so the causal relationships between variables could not be established. More longitudinal studies may be needed to discuss causality between these variables.

Third, we were not able to determine which children received online learning without parental supervision due to the questionnaire being filled out by parents. Future research could focus on children that need to perform online learning without parental supervision. In addition, the interaction between children and parents and teachers may be considered as a new issue of future study.

Finally, a limited number of variables were discussed in this study. Some other factors may also have an impact on online learning effectiveness, such as students’ affinity to online technologies, the feelings of isolation due to lockdown, and mental state (e.g., [3,12,68]). To enhance objectivity, future studies could consider taking other approaches to measuring online learning effectiveness. For example, the effectiveness of online learning can be further evaluated by students’ actual performance (e.g., grades, the score of assignments) [69,70,71].

## Figures and Tables

**Table 1:** Reliability and Validity Analysis.

Variables	Cronbach’s α	CR	AVE	FL
PATOL (6 items)	0.946	0.947	0.781	0.647–0.878
PSE (4 items)	0.852	0.859	0.607	0.640–0.860
POLI (5 items)	0.925	0.930	0.691	0.845–0.921

**Table 2:** Descriptive statistics of model variables and correlations among model variables.

Variables	*M*	*SD*	PATOL	PSE	POLI
PATOL	4.098	0.739	-		
PSE	2.839	0.462	0.525 **	-	
POLI	3.312	1.046	−0.135 **	−0.132 **	-

Note: ** *p* < 0.01.

**Table 3:** Regressions testing PSE as a mediator in the relationship between PATOL and POLI.

Regression Equations. (N = 18,170)		*β*	*SE*	*t*	*R* ^2^	*F*
Outcome:	Predictor:					
POLI					0.02	98.48 ***
	gender	0.056	0.017	3.249 **		
	age	−0.056	0.010	−5.428 ***		
	LOL	−0.005	0.007	−0.671		
	PATOL	−0.192	0.011	−18.395 ***		
PSE					0.28	1743.94 ***
	gender	0.019	0.007	2.919 *		
	age	−0.020	0.004	−4.944 ***		
	LOL	0.007	0.003	2.461 *		
	PATOL	0.326	0.004	82.288 ***		
POLI					0.03	100.13 ***
	gender	0.060	0.017	3.479 **		
	age	−0.060	0.010	−5.814 ***		
	LOL	−0.003	0.007	-0.486		
	PSE	−0.199	0.020	−10.222 ***		
	PATOL	−0.127	0.012	−10.419 ***		

Note: LOL = length of online learning, LLCI = lower limit confidence interval, ULCI = upper limit confidence interval. The PATOL, PSE, and POLI in regression models were standardized. * *p* < 0.05, ** *p* < 0.01, *** *p* < 0.001.

**Table 4:** The result of direct effect, indirect effect, and total effect.

	Effect	*SE*	LLCI	ULCI
Direct effect	−0.127	0.012	−0.151	−0.103
Indirect effect	−0.065	0.007	−0.079	−0.052
Total effect	−0.192	0.011	−0.213	−0.172

Note: LLCI = lower limit confidence interval, ULCI = upper limit confidence interval.

## Data Availability

Some or all data and models that support the findings of this study are available from the corresponding author upon reasonable request.
